# Apparent Long-Term Cure Following Conversion Therapy for Initially Unresectable Pancreatic Ductal Adenocarcinoma: A Case Report

**DOI:** 10.7759/cureus.105912

**Published:** 2026-03-26

**Authors:** Evangelia Florou, Yoh Zen, Parthi Srinivasan, Andreas Prachalias

**Affiliations:** 1 Hepato-Pancreato-Biliary Surgery, King's College Hospital, London, GBR; 2 Pathology and Laboratory Medicine, King's College Hospital, London, GBR; 3 Hepato-Pancreato-Biliary Surgery and Liver Transplantation, London Bridge Hospital, London, GBR

**Keywords:** conversion therapy, folfirinox, locally advanced pancreatic cancer, long-term survival, pancreatic ductal adenocarcinoma, pathological complete response

## Abstract

Locally advanced pancreatic ductal adenocarcinoma (LA-PDAC) is increasingly managed with neoadjuvant chemotherapy with the aim of tumour downstaging and potential conversion to resection. LA-PDAC is typically defined by tumour involvement of major vascular structures precluding upfront surgical resection in the absence of distant metastases. However, only a minority of patients ultimately undergo secondary surgery, and pathological complete response (pCR) remains rare.

A 64-year-old male was diagnosed in 2011 with LA-PDAC involving the pancreatic neck with vascular involvement precluding upfront resection. He received systemic chemotherapy with capecitabine-cisplatin, followed by four cycles of FOLFIRINOX (oxaliplatin, irinotecan, leucovorin, and 5-fluorouracil). Restaging imaging demonstrated marked tumour regression without metastatic disease. In December 2012, he underwent total pancreatectomy and splenectomy. Histopathological examination revealed complete tumour regression (ypT0N0R0). The patient remained free of pancreatic cancer recurrence for over 13 years and ultimately died from an unrelated primary lung malignancy.

Although pCR following neoadjuvant therapy for PDAC is uncommon and associated with improved survival, long-term disease-free survival beyond a decade after conversion surgery for initially unresectable disease is rarely reported. This case highlights the potential impact of tumour biology, sustained systemic response, and multidisciplinary reassessment in selected patients with LA-PDAC.

## Introduction

Pancreatic ductal adenocarcinoma (PDAC) remains a highly lethal malignancy, with approximately 30-40% of patients presenting with locally advanced disease characterised by vascular involvement precluding upfront resection [1,2]. Historically managed with palliative intent, the management paradigm for locally advanced PDAC (LA-PDAC) has evolved with the introduction of effective multi-agent chemotherapy regimens.

FOLFIRINOX, a combination regimen of oxaliplatin, irinotecan, leucovorin, and 5-fluorouracil, has emerged as one of the most active systemic therapies in this setting, demonstrating improved response rates and survival in patients with advanced pancreatic cancer [3,4]. FOLFIRINOX-based treatment has enabled tumour downstaging in selected patients, allowing secondary resection in 10-30% of cases [3,4]. Patients undergoing successful conversion to surgery demonstrate significantly improved survival compared with those managed non-operatively [5,6]. However, pathological complete response (pCR) following neoadjuvant therapy remains rare, occurring in fewer than 5% of resected cases [7,8].

While pCR is associated with favourable outcomes, long-term disease-free survival exceeding a decade following conversion surgery for initially unresectable PDAC has rarely been documented [9-11]. We report a case of LA-PDAC treated with multimodal systemic therapy followed by resection, achieving a complete pathological response and remaining disease-free for over 13 years. This case highlights the potential impact of tumour biology and appropriate patient selection in the era of conversion therapy.

## Case presentation

Initial presentation and diagnosis

**Figure 1:**
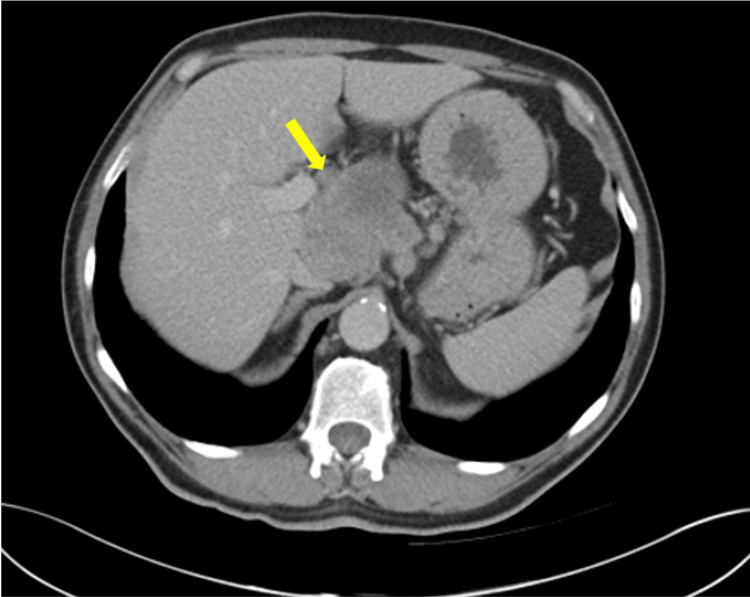
Contrast-enhanced axial CT at diagnosis showing a pancreatic mass centred in the neck of the pancreas (yellow arrow).

**Figure 2:**
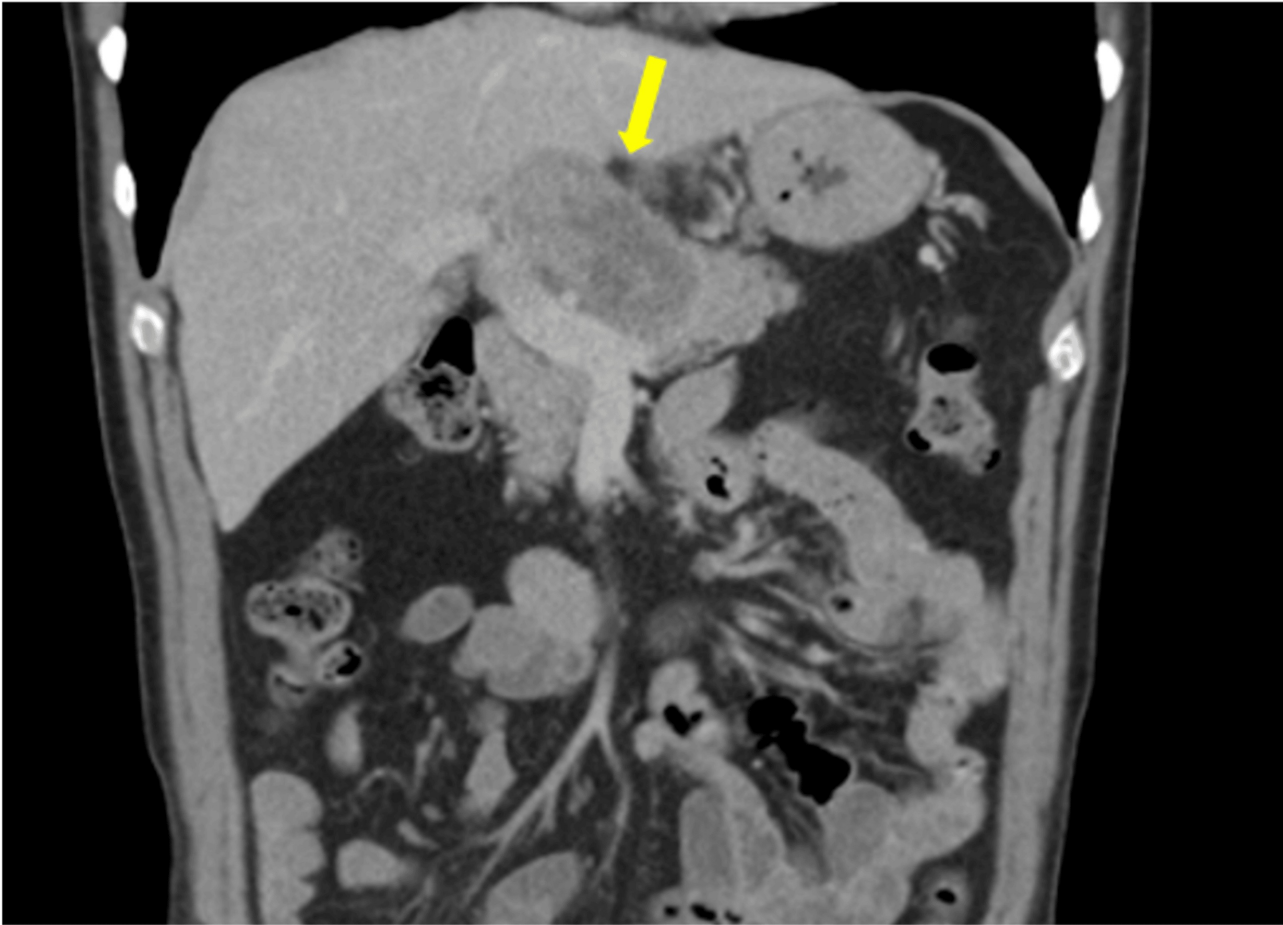
Coronal CT image at presentation demonstrating the primary tumour in the pancreatic neck (yellow arrow) encasing the hepatic artery, consistent with locally advanced disease.

A 64-year-old male presented to a local hospital and was diagnosed in May 2011 with LA-PDAC, measuring 96 × 49 mm and centred in the pancreatic neck. Imaging demonstrated abutment of the portal vein and encasement of the common hepatic artery (Figures 1, 2). There was no evidence of distant metastasis on staging investigations. He was subsequently referred to the local hospital during the course of treatment.

Initial endoscopic ultrasound (EUS)-guided sampling demonstrated malignant epithelial cells suspicious for PDAC. Due to limited cytological material, a subsequent CT-guided core biopsy was performed, confirming atypical epithelial cells consistent with adenocarcinoma. At diagnosis, serum CA19-9 was 28 kU/L (reference range = 0-35 kU/L), indicating a non-secretory tumour phenotype. Consequently, CA19-9 was not used as a serial biomarker during treatment.

Neoadjuvant and systemic therapy

The patient commenced combination chemotherapy with capecitabine and cisplatin, achieving an initial radiological response. At the time of diagnosis in 2011, capecitabine- and platinum-based regimens were utilised in selected European centres for LA-PDAC, particularly prior to the widespread adoption of FOLFIRINOX (oxaliplatin, irinotecan, leucovorin, and 5-fluorouracil).

In February 2012, follow-up imaging raised suspicion of liver metastases, prompting escalation to FOLFIRINOX. However, subsequent cross-sectional imaging, including MRI, did not confirm the presence of liver lesions, and the initial findings were ultimately interpreted as an imaging artefact. No hepatic metastases were identified on imaging ever after.

He received four cycles, with intermittent delays due to treatment-related toxicities, completing therapy in September 2012.

During systemic treatment, adverse effects were manageable. Capecitabine was associated with hand-foot syndrome requiring temporary interruption and pyridoxine support. Following transition to FOLFIRINOX, the patient experienced irinotecan-related diarrhoea responsive to atropine, intermittent severe diarrhoea necessitating dose reduction, and one episode of neutropenic sepsis managed with granulocyte colony-stimulating factor (G-CSF). Despite these toxicities, overall tolerance was acceptable.

**Figure 3:**
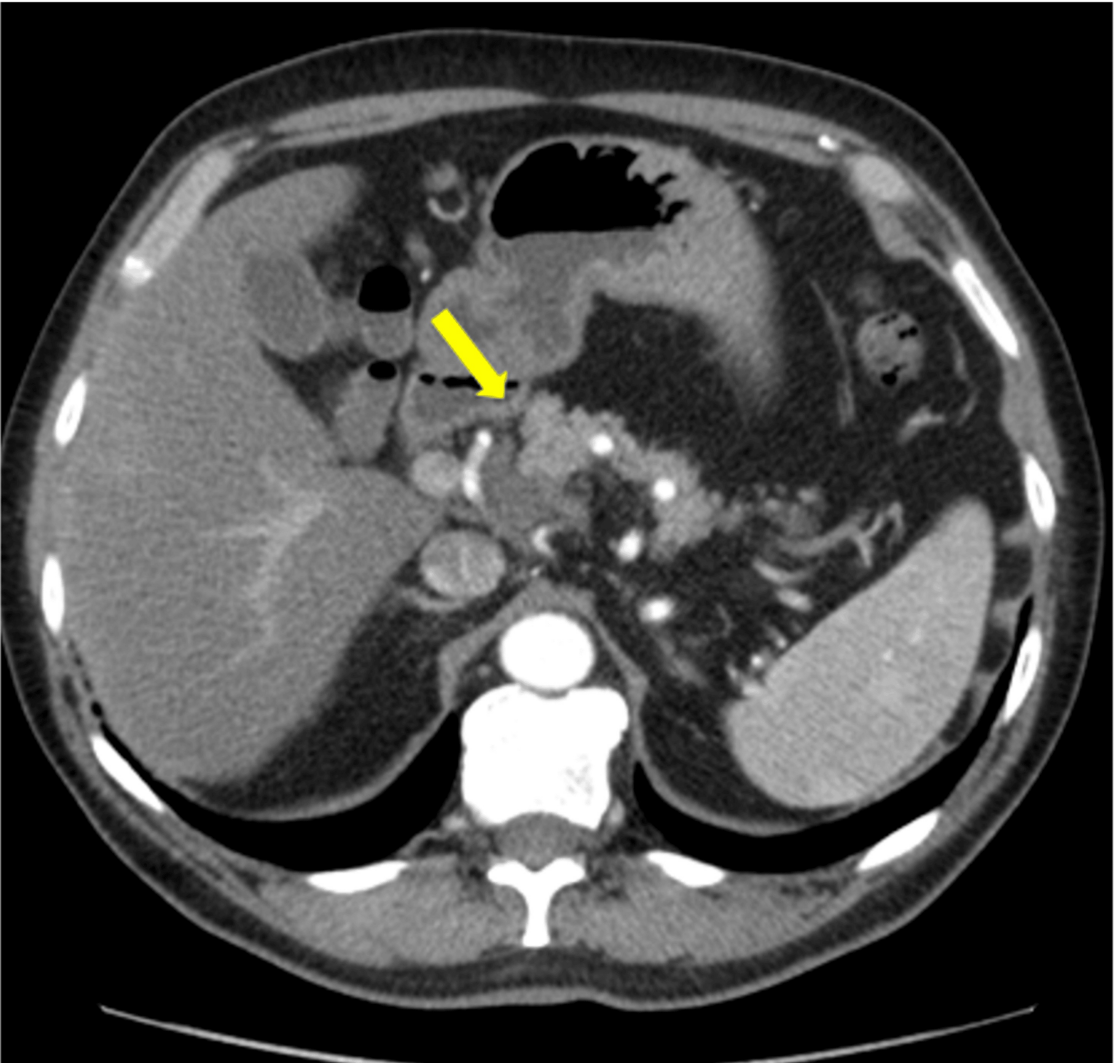
Axial contrast-enhanced CT following chemotherapy showing marked reduction of the soft-tissue density around the common hepatic artery (yellow arrow), consistent with excellent response of the primary tumour.

**Figure 4:**
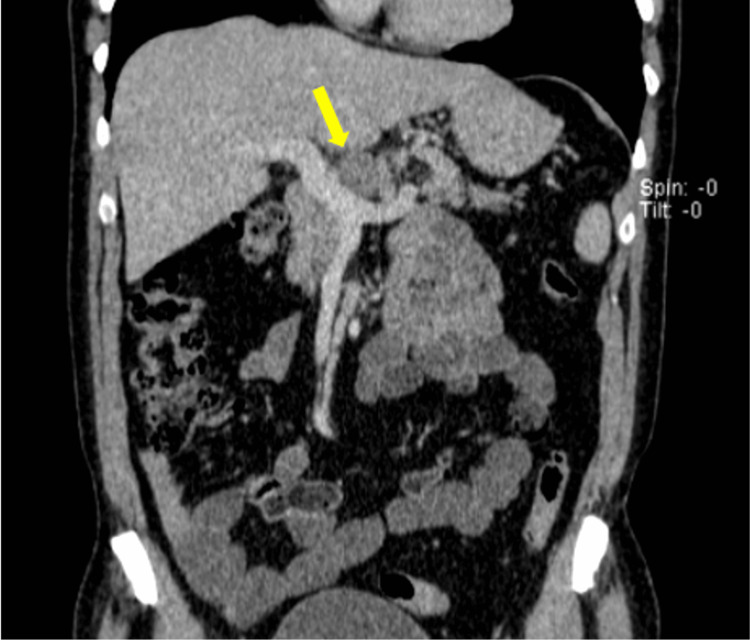
Coronal contrast-enhanced CT following chemotherapy showing a marked reduction in size of the primary pancreatic lesion (yellow arrow), indicating excellent treatment response.

Restaging CT demonstrated marked radiological response, with significant reduction in tumour volume and no evidence of distant metastasis (Figures 3, 4). The case was discussed at the multidisciplinary team (MDT) meeting at our tertiary hepatopancreatobiliary (HPB) centre and was deemed potentially resectable.

Surgical management and pathology

**Figure 5:**
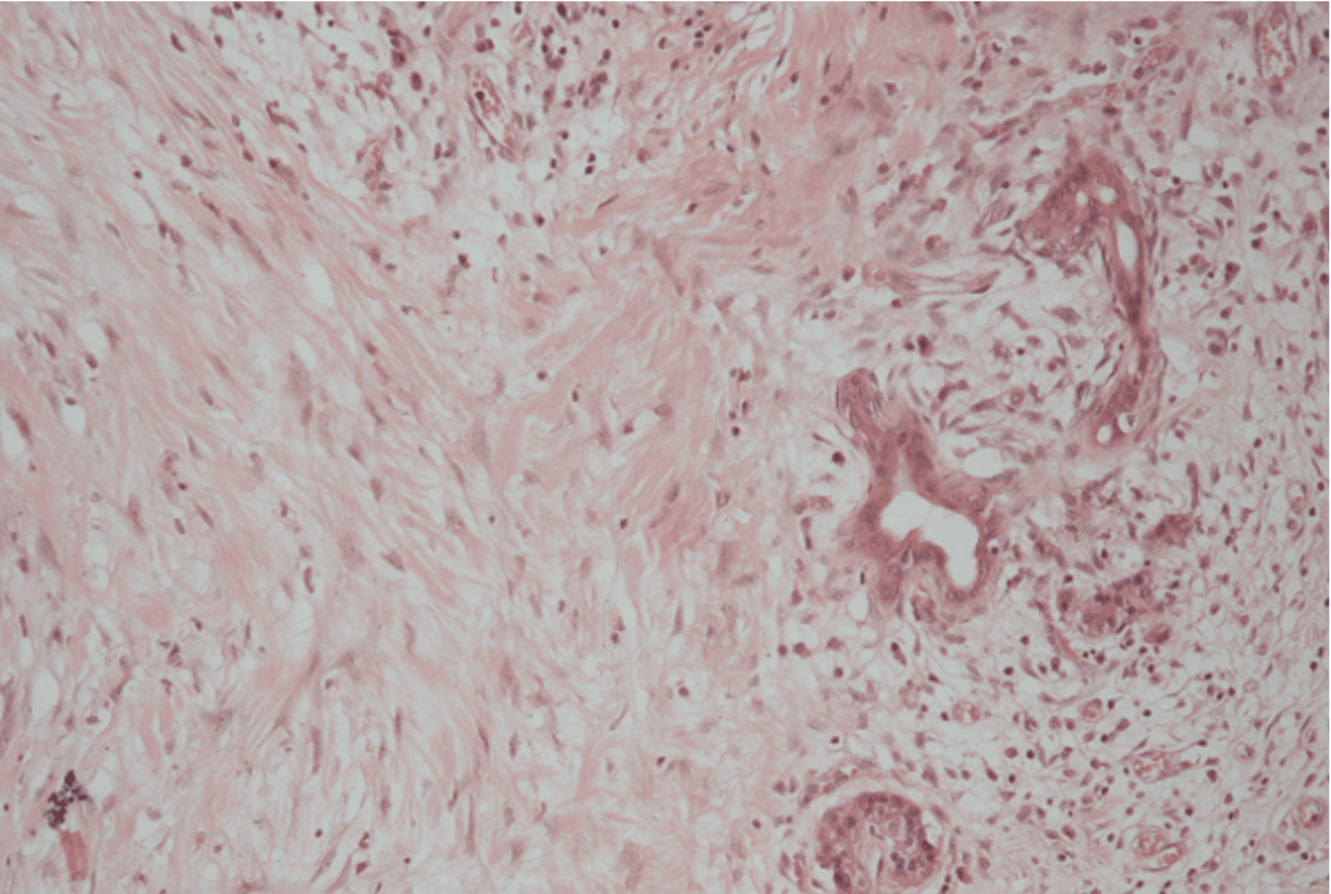
Haematoxylin and eosin-stained section (x200 magnification) of the resected specimen showing therapy-related dense fibrosis with residual benign ducts and islets, with no viable tumour identified, consistent with complete pathological response (ypT0N0R0).

In December 2012, the patient underwent total pancreatectomy and splenectomy. The postoperative course was uneventful. Histopathological assessment was performed according to the College of American Pathologists (CAP) protocol for pancreatic cancer resection specimens [12]. The specimen was fixed in 10% neutral buffered formalin, routinely processed, and embedded in paraffin. Sections were stained with haematoxylin and eosin using standard methods. Adequate sampling was confirmed through multiple representative sections from the tumour bed and surrounding parenchyma. Examination demonstrated extensive therapy-related fibrosis with residual benign ducts and islets, without viable invasive carcinoma. No lymph node involvement was identified (0/6). Findings were consistent with a CAP grade 0 complete pathological response (ypT0N0R0) (Figure 5).

Postoperative follow-up

**Figure 6:**
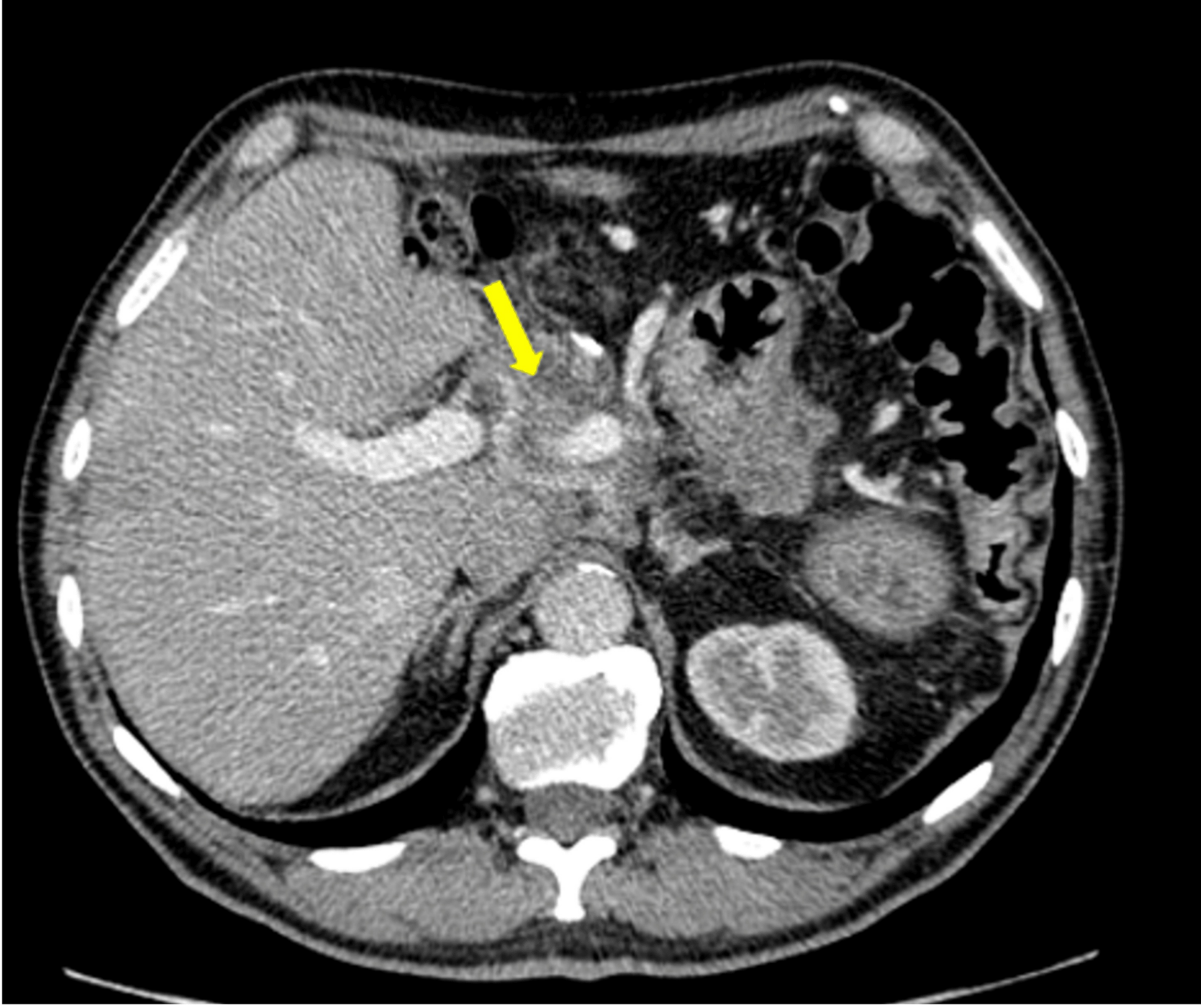
Axial CT performed in 2013 showing subtle soft-tissue density around the celiac axis, initially raising concern for recurrence (yellow arrow). Subsequent serial imaging confirmed stability of these postoperative changes with no radiologic, clinical, or biochemical evidence of disease recurrence.

The patient was referred back to his local oncology service for surveillance. Cross-sectional imaging was performed every six months for five years. In 2013, soft-tissue density around the coeliac axis raised suspicion of recurrence; however, serial imaging and comparative assessment supported postoperative change (Figure 6). No clinical, biochemical, or radiological evidence of recurrence was observed. After five years of disease-free surveillance, the patient was discharged from routine oncology follow-up in July 2018.

Second primary malignancy and death

**Figure 7:**
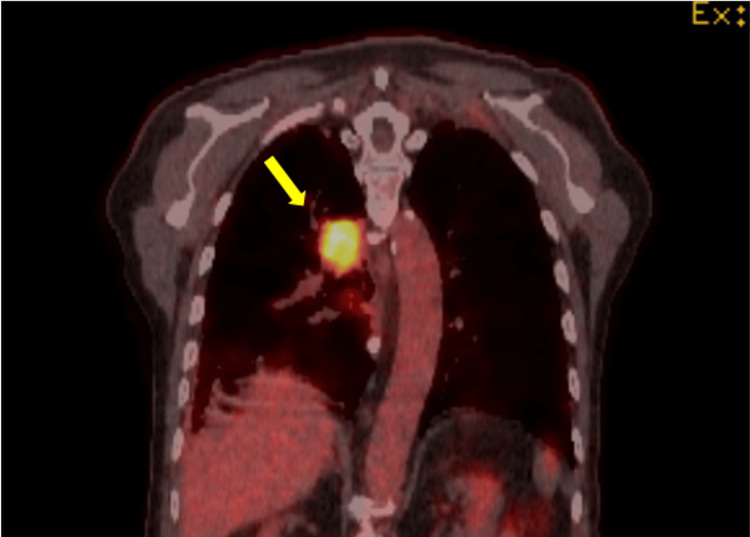
FDG PET-CT demonstrating a metabolically active lesion in the right lung (yellow arrow), consistent with a new primary pulmonary malignancy. FDG PET-CT: fluorodeoxyglucose positron emission tomography combined with computed tomography.

In February 2025, the patient was re-referred to oncology with dyspnoea and weight loss. CT imaging revealed a large pulmonary mass with mediastinal nodal involvement and bone metastases, consistent with primary lung carcinoma (T4N2M1b) (Figure 7). The radiological pattern, characterised by a dominant lung lesion with intrathoracic spread, was not typical of metastatic pancreatic adenocarcinoma. Given advanced-stage disease and comorbidities including emphysema, prior cerebrovascular accident, and hypertension, tissue biopsy was deferred. He received palliative gemcitabine and carboplatin chemotherapy and radiotherapy for painful rib metastases. The patient died in March 2025, 13 years and 10 months after initial diagnosis, having remained free of pancreatic cancer recurrence throughout his life.

## Discussion

LA-PDAC represents a challenging subgroup of pancreatic cancer, accounting for approximately 30-40% of newly diagnosed cases [1,2]. With modern systemic therapy, 10-30% of patients may achieve sufficient tumour regression to permit secondary resection following neoadjuvant chemotherapy, most commonly with FOLFIRINOX-based regimens [3,4]. Patients undergoing successful conversion to surgery demonstrate improved survival compared with those managed non-operatively [5,6]. In large series, median overall survival following conversion surgery ranges from approximately 25 to 40 months [5,6].

pCR in this setting remains rare, occurring in fewer than 5% of resected cases [7,8]. However, pCR has been consistently associated with significantly prolonged survival [7,8]. Reported median disease-free survival in patients achieving pCR approaches 34 months, with overall survival extending beyond 60 months in multicentre series [7,8]. Despite this favourable prognostic association, recurrence has been reported in up to 40-50% of patients achieving pCR in contemporary series [8], indicating that pCR cannot be regarded as synonymous with cure.

At the time of this patient’s diagnosis in 2011, FOLFIRINOX had only recently been introduced following publication of the PRODIGE 4/ACCORD 11 trial, which demonstrated a significant survival advantage over gemcitabine in metastatic pancreatic cancer [13]. Its incorporation into neoadjuvant and conversion strategies for LA-PDAC occurred subsequently. Earlier European practice in selected centres included capecitabine- and platinum-based regimens prior to widespread adoption of FOLFIRINOX [1,13]. Escalation to FOLFIRINOX in this case, therefore, reflected contemporaneous standards of care.

FOLFIRINOX is now considered the most active systemic regimen for fit patients with advanced PDAC and has demonstrated substantial tumour downstaging potential [3,4,14]. Contemporary international data suggest improved survival among patients undergoing induction FOLFIRINOX prior to resection compared with alternative regimens [15]. Although this patient received sequential chemotherapy rather than upfront FOLFIRINOX, it is likely that the latter regimen was the principal driver of tumour regression and eventual pCR.

The development of a primary lung carcinoma more than 13 years after completion of systemic therapy is unlikely to be treatment-related. FOLFIRINOX is not known to predispose to solid tumour induction, and therapy-related secondary malignancies typically occur with shorter latency and most commonly involve haematological cancers [11]. Long-term survivors of pancreatic cancer may develop second primary malignancies independent of prior treatment exposure [11].

We acknowledge several limitations. Histological material from the initial diagnostic biopsy and macroscopic specimen photographs were not available in the institutional archive. Additionally, the lung lesion was not biopsied; however, the radiological pattern and prolonged disease-free interval strongly supported a de novo primary malignancy rather than metastatic pancreatic recurrence.

Long-term survival exceeding 10 years following resection for PDAC remains rare and is predominantly reported in patients with initially resectable disease [9-11]. Multi-institutional analyses report 10-year survival rates below 5-7% [9,10,13]. Paniccia et al. [9] and Dal Molin et al. [10] demonstrated that such long-term survivors typically exhibit biologically favourable features, including node-negative disease, absence of vascular invasion, and low preoperative CA19-9 levels. Seppänen et al. [16] similarly reported that long-term survival was confined to highly selected patients achieving R0 resection. Notably, patients with arterial invasion or distant disease rarely achieved decade-long survival, underscoring the dominant role of intrinsic tumour biology. Similar exceptional responses have been reported, albeit rarely, in metastatic PDAC [17].

This case, therefore, represents an exceptionally favourable biological response within the context of initially unresectable disease. Sustained disease control, complete histological regression (ypT0N0R0), and absence of recurrence over 13 years suggest profound chemosensitivity and favourable tumour biology.

In conclusion, this case illustrates that durable long-term disease-free survival following conversion therapy and resection for initially unresectable LA-PDAC is possible in highly selected patients. While pCR cannot be equated with a cure, this report supports the continued role of multidisciplinary reassessment and aggressive systemic therapy in carefully selected individuals.

## Conclusions

PDAC remains one of the most lethal malignancies, with a large proportion of patients presenting with LA or metastatic disease that historically precluded curative treatment. However, the therapeutic landscape has evolved significantly over the past decade with the introduction of modern multi-agent chemotherapy regimens such as FOLFIRINOX, which have improved systemic disease control and enabled tumour downstaging in selected patients. These advances have expanded the role of multimodal management and conversion surgery, offering a potential pathway to long-term survival in carefully selected individuals with initially unresectable disease.

Despite these advances, pCR and prolonged disease-free survival remain rare in pancreatic cancer. Exceptional cases such as the present report highlight the profound biological heterogeneity of pancreatic ductal adenocarcinoma and underscore the central role of tumour biology in determining therapeutic response and prognosis. The durable remission observed in this patient, exceeding 13 years after conversion therapy and resection, represents an extraordinary outcome within the existing literature. Recognition and investigation of such rare responders may provide valuable insights into the biological determinants of treatment sensitivity and help refine future strategies aimed at identifying patients most likely to benefit from aggressive multimodal therapy.
